# The effects of betulinic acid chronic administration on the motor, non-motor behaviors, and globus pallidus local field potential power in a rat model of hemiparkinsonism

**DOI:** 10.22038/IJBMS.2022.65623.14434

**Published:** 2022-11

**Authors:** Maryam Abrishamdar, Alireza Sarkaki, Yaghoob Farbood

**Affiliations:** 1 Persian Gulf Physiology Research Center, Medical Basic Sciences Research Institute, Ahvaz Jundishpur University of Medical Sciences, Ahvaz, Iran; 2 Department of Physiology, Medicine Faculty, Ahvaz Jundishapur University of Medical Sciences, Ahvaz, Iran

**Keywords:** Betulinic acid, Catalase, Hemiparkinsonism, Local EEG, Stride-length

## Abstract

**Objective(s):**

Parkinson’s disease (PD) is a neurodegenerative disorder involving the central nervous system associated with motor and non-motor impairments. Betulinic acid (BA) is a natural substance considered an antioxidative agent. This study aimed to investigate the therapeutic potential of BA on motor dysfunctions and globus pallidus (GP) local EEG power in a 6-hydroxydopamine (6-OHDA)-induced rat model of hemiparkinsonism.

**Materials and Methods:**

Adult Wistar rats were categorized into different groups, containing; Sham, PD, and treated groups including different doses of BA (0.5, 5, and 10 mg/kg, IP), and L-dopa (20 mg/kg, PO, as positive control). The lesion was induced in the right medial forebrain bundle by injection of 6-OHDA (20 µg/kg). The treatment was begun just after the approved rotational test induced by apomorphine, 14 days after 6-OHDA administration. Motor behaviors such as catalepsy and stride-length and non-motor responses, including GP local EEG, were then assessed. Also, the levels of GSH, catalase, and concentration of dopamine in the brain tissue were measured.

**Results:**

Treatment of hemiparkinsonian rats with BA significantly improved catalepsy and stride-length (*P*<0.001 and *P*<0.01, respectively) and GP frequency bands’ powers (*P*<0.001). Moreover, the activities of GSH (*P*<0.001), catalase (*P*<0.001), and the concentration of dopamine (*P*<0.001) in the brain were increased.

**Conclusion:**

Current results proved the potent ability of BA to scavenge free radicals and to remove oxidative agents in the brain tissue. This natural product could be considered a possible therapeutic compound for motor and non-motor disorders in PD.

## Introduction

Parkinson’s disease (PD) is considered the second most common central nervous system (CNS)-associated degenerative disorder, characterized by motor symptoms including rigidity, bradykinesia, tremor, and postural reflex disturbances (1). Also, a series of non-motor symptoms, such as sleep complaints, constipation, olfactory dysfunction, various neuropsychiatric manifestations, cognitive deficiency, and dementia, was reported in primary and progressive courses of disease (2). 

The pathophysiological mechanism of PD could be associated with a decrease in the striatum’s dopamine (DA) levels, causing an elevation in the activity of the globus pallidus internal segment (GPi) and a tonic over inhibition of the pallidal-recipient thalamic relay cells, which results in a thalamocortical dysrhythmia interaction followed by a significant generation of low threshold calcium spikes and an increase in some frequency electroencephalographic (EEG) power in the thalamus (3). EEG activity is subdivided into different frequency bands, including, gamma, beta, alpha, theta, and delta. Changes in alpha band expression have been hypothesized to underlie some cognitive and attentive difficulties observed in PD patients. Also, the gamma band has been related to sensory, cognitive processing, attention, long-term memory, and language tasks. Eventually, the delta band is associated with sleep functions **(**4**)**. Oscillations in the beta and gamma frequencies play a major role in motor control in the basal ganglia **(**5, 6**)**. Theta band plays a role in processing conflict-related signals that include freezing gaits and is strongly aligned with previous neuroimaging studies of freezing **(**7**)**. Genetic susceptibility, mitochondrial dysfunction, oxidative stress, immunodeficiency, excitotoxicity, apoptosis, and deficient neurotrophic support are the most important risk factors associated with neuronal degeneration in PD patients (8).

Production of reactive oxygen species (ROS) due to DA oxidation and abnormal generation of ROS leads to oxidative stress and neuronal death (9). In addition, previous studies have shown that exposure to oxidative stress can result in neuronal cell injury in PD patients (10). As an important protective mechanism, the glutathione (GSH) system removes free radicals to minimize oxidative stress and maintains protein thiols in their appropriate redox state. Moreover, antioxidant enzymes, such as catalase (CAT), are important mediators in reducing oxidative stress molecules, including hydroxyl radicals’ increase (11), Thus, a high antioxidant activity has been correlated with protection against neurodegenerative disorders (12).

Among the different animal models, administering 6-hydroxydopamine (6-OHDA) in the unilateral striatal is one of the best-known procedures to induce the pathological, behavioral, and biochemical manifestations of PD. These are attributed to the formation of different oxidant agents, lipid peroxidation (LPO), depletion of reduced GSH, and mitochondrial deficits (8).

In recent years, studies have been focused on medicinal plants-derived antioxidant agents against free radicals, including reactive oxygen species (ROS) and reactive nitrogen species (RNS) (13). Recently, betulinic acid (BA), as a white birch tress-derived pentacyclic triterpene, has been introduced as an interesting antioxidant agent to inhibit mitochondrial impairment that is associated with oxidative stress and may be used in the modulation of a wide variety of acute and chronic conditions (14). Importantly, BA can cross the blood-brain barrier, making it an interesting target for treating neurodegenerative disorders (15).

Hence, based on the information mentioned above, the current study evaluated the potential efficacy of BA chronic administration on motor dysfunction and frequency bands’ power GP local field potential EEG after Hemi PD induction in rats.

## Materials and Methods

***Animals***

Adult male Wistar rats (10–12 weeks old, 270–320 g) were purchased from Central Animal Lab, Ahvaz Jundishapur University of Medical Sciences (AJUMS) (Ahvaz, Iran). Animals were kept in a 12/12 hr light-dark cycle, and treatment procedures were conducted during the light phase (08:00 am to 2:00 pm). Also, all experiments were approved by the Ethics Committee of Ahvaz Jundishapur University of Medical Sciences (IR.AJUMS.REC.1396.662). 

***Experimental design***

Forty-eight rats were randomly categorized into five different groups (n=8), including:

1) Sham animals; received a vehicle of 6-OHDA (Sigma-Aldrich Chemical Co., St. Louis, MO, USA) 0.01% ascorbic acid-contained normal saline into the right medial forebrain bundle (MFB).

2) Hemi-PD+Veh (Vehicle), MFB lesioned rats, received 20 µg/4 µl 6-OHDA **(**16**)** into the right MFB, and 14 days after stereotaxic surgery started treating with the vehicle of BA (Sigma-Aldrich Chemical Co., St. Louis, MO, USA) (DMSO-contained normal saline (Alfasan Co., Woerden, Netherland) intraperitoneally for 7 days).

3-5) Hemi-PD+BA groups, MFB-lesioned rats, received BA 0.5, 5, and 10 mg/kg, 14 days after stereotaxic surgery, and the treatment lasted up to 7 days **(**17**)**. 

6) Hemi-PD+LD as positive control, MFB-lesioned animals received LD (20 mg/kg, PO), which was administered 14 days after stereotaxic surgery and lasted up to 7 consecutive days (18).

***Surgical procedures for 6-OHDA injections and electrode implantation***

Recently, lesioning the DA axon terminals in the MFB following the catecholamine-selective neurotoxin -6-OHDA- injection was introduced as a novel method to induce rat models that are partially lesioned in the nigrostriatal DA system (16). Before the stereotaxic lesion of MFB and 30 min before the delivery of 6-OHDA, rats received 25 mg/kg, IP desipramine (Sigma-Aldrich Chemical Co., St. Louis, MO, USA) to prevent damage to the noradrenergic neurons. Then, they were anesthetized using IP administration of ketamine (70 mg/kg)/xylazine (4 mg/kg) (Alfasan Co., Woerden, Netherland) (19). Anesthetized rats were transferred into the stereotaxic apparatus (Narishige, Japan), and their heads were fitted. 6-OHDA (20 µg/3µl of 0.02% ascorbic acid-contained normal saline) or its vehicle (in the sham group) was injected into the right MFB using a Hamilton syringe at a rate of 0.2 µl/min for 10 min duration before being drawn back at a rate of 2 mm/min (20), according to the coordinates in Paxinos and Watson atlas: AP: -4.4 mm, ML: +1.3 mm from bregma and DV: -8.4 mm from skull surface (21).

Following the MFB lesion and to record a local EEG of the GP nucleus, a bipolar stainless steel wire recording electrode (stainless steel Teflon coated 0.005” bare 0.008” coated, A-M systems, Inc. WA, USA) was implanted in the left GP at AP: -1.3 mm (from bregma) ML: 3.2, DV: -6.5 mm (from skull surface) and was lowered into the brain so that the active pole (taller wire) of an electrode was placed in the left GP nucleus (3). The electrode was fixed on the skull with small stainless steel screws and dental acrylic, and the incision was sutured (20).

***Apomorphine-induced circling behavior***

Apomorphine-induced rotational behavior was implicated in assessing the motor imbalance due to the unilateral lesion of the MFB. First, for 10 min, animals were adapted to their environment before turns contralateral to the lesion were recorded over 30 min (22). Next, to deplete the nigrostriatal system from dopamine, they received subcutaneous apomorphine hydrochloride (0.5 mg/kg) (Sigma-Aldrich Chemical Co., St. Louis, MO, USA) suspended in 0.02% ascorbic acid-containing normal saline. Fourteen days following the lesion and before every treatment, all animals were evaluated for rotational behavior (23).

***Treatment***

Sham and hemi PD + Veh animals were injected IP DMSO (20% DMSO in normal saline). BA has been suspended in 20% DMSO solution (24) and prepared in 0.5, 5, and 10 mg/kg doses for different treated groups. The hemi PD+LD group received oral levodopa/carbidopa (4:1) (Sigma-Aldrich Chemical Co., St. Louis, MO, USA) dissolved in normal saline through gavage at a 20 mg/kg dosage (18). The treatment procedure was conducted for seven consecutive days in all groups.

***Motor assessment***

*Morpurgo’s test for muscle stiffness*

The scoring of muscle rigidity was adopted according to a three-step evaluation as follows: 

Step 1; The animal did not move when placed on a flat table, but following a mild touch, it moved and was assigned a score of 0.5.

Step 2; One of the front paws was put on a 3 cm high woody block; if the animal did not correct the position for 10 sec, a score of 0.5 was assigned. Accordingly, the second paw was put and scored as mentioned. 

Step 3; One of the front paws was put on a 9 cm high woody block, while the other was left hanging. A positive catatonic reaction was gauged by the failure to modify the status for 10 sec and assigned a score of 1. Similarly, the process was conducted for the other paw. Thus, if the animal showed full catatonia, it was allocated a total score of 3.5 (25).

*Forepaw stride length during walking*

The forepaw of trained rats was placed in black ink and normally walked straight. Then, the length of the forepaw steps was measured. The distance between each step on the same side of the body from the top of the toe print of the one step to the same point of the latter step was measured to determine the stride length. To this, the stride length of four clear steps was quantified and an average was reported (26).

***Electrophysiological recording***

To record the local EEG from the right GP nucleus, four channels Powerlab, LabChart software version 7, and ML 135 bio amplifier (AD Instruments, Australia) with 1 mV amplification, sample recording 400 HZ, and 0.3-70 Hz bandpass filtration for 5 sec was applied in free-moving animals. The crude EEG and its alpha, beta, gamma, delta, and theta frequency bands’ mean powers within three 5-second courses were evaluated and compared between different groups. The electrical power of frequency bands was measured as μV2/Hz (27).

***Oxidative parameters & dopamine assay***

As the final step of the study, animals were sacrificed after deep and irreversible anesthesia using 100 mg/kg sodium thiopental (nesdonal), intraperitoneally. They were sacrificed rapidly, and the brain was rapidly detached from the skull, washed with cold saline, and frozen at -80 °C until further examinations. To measure CAT, GSH, and DA levels, first a 10% homogenate suspension was prepared. To do this, 200 mg of brain tissue was homogenized in a cold 1.5% KCl solution. Then, a Biochemical ELISA kit (Zellbio Co., Germany) was applied based on the ‘manufacturer’s instructions (28).

***Statistical analysis***

Data analysis was performed using the GraphPad Prism software (version 7, GraphPad Software Inc., San Diego, USA), expressed as means ± SEM, and was analyzed statistically by one-way ANOVA, followed by Tukey’s *post hoc* test. *P*<0.05 was considered statistically significant.

## Results

***Apomorphine-induced circling test***

**Figure 1:**
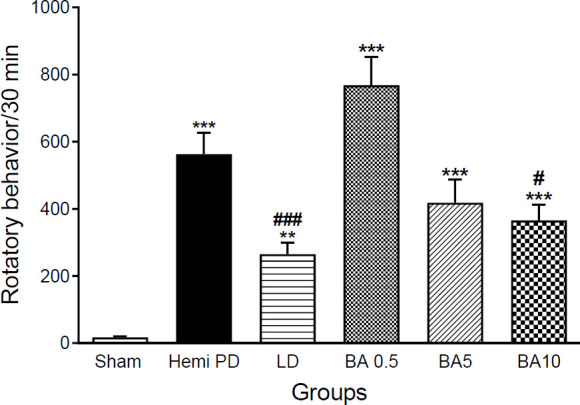
Results of rotatory behavior following BA and LD treatments in different groups. Data were shown as mean ± SEM (n=8) and analyzed using one-way ANOVA and Tukey's *post hoc* tests to compare different groups, **P<0.01 & ***P<0.001, compared with the sham group. #*P*< 0.05 & ###*P*<0.001 vs hemi PD group three weeks after surgery. (Hemi PD= lesioned animals, LD=L-Dopa 20 mg/kg treatment group, BA0.5, BA5, BA10 = Betulinic acid 0.5, 5 & 10 mg/kg treated groups)

As shown in Figure 1, 21 days after surgery (7 days after treatment), apomorphine-induced contralateral rotation was dramatically elevated in 6-OHDA-lesioned animals in comparison with the sham group (*P*<0.001). Also, seven days following treatments, the frequency of contralateral turns in treated animals (LD 20 mg/kg & BA 10 mg/kg) was significantly decreased in comparison with the hemi PD group (*P*<0.001 & *P*<0.05, respectively), F (5, 36) =27.4, while no significant difference was reported between BA (5 mg/kg), BA (0.5 mg/kg) treated, and hemi PD groups (*P>0.05*). 

***Catalepsy***

**Figure 2:**
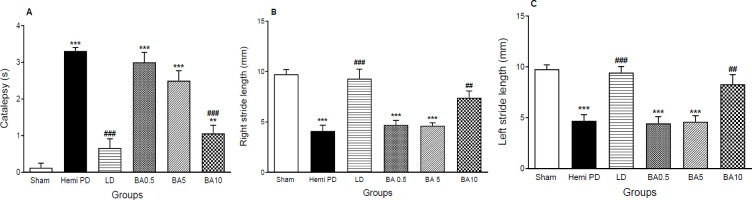
Effects of LD & BA treatment on catalepsy (A) and stride length (B & C) in different groups. Data were shown as mean ± SEM (n=8) and analyzed using one-way ANOVA and Tukey’s *post hoc* tests to compare various groups, **P<0.01 & ***P<0.001, compared with the sham group, ##*P*<0.01 & ###*P*<0.001 compared with hemi PD group, three weeks after surgery. (Hemi PD=Lesioned animals, LD=L-Dopa 20 mg/kg, BA0.5, BA5, BA10 = Betulinic acid 0.5, 5 & 10 mg/kg treated groups)

As measured by Morpurgo’s test (Figure 2A) 2 weeks post-surgery, hemi PD rats displayed a progressive increase in the descent latency in comparison with the control group (*P*<0.001). The results showed that 7 days of treatment of hemi PD rats with BA, at doses of 0.5 and 5 mg/kg, could not reduce catatonic responses in comparison with the sham-treated group while 10 mg/kg dosage of BA and 20 mg/kg of LD induced a prominent reduction in catalepsy (*P*<0.001), F (5,33) =24.7, while complete inhibition was not resulted.

***Forelimb stride length***

As shown in (Figure 2B & 2C), *post hoc* analysis showed that unilateral lesioning of MFB resulted in a meaningful forelimb walking length in the hemi PD group (*P*<0.001). No changes were acquired after Treatment with BA (0.5 and 5 mg/kg), but then it was remarkably reversed by BA10 mg/kg (*P*<0.01) and LD (*P*<0.001). Right Stride Length; F (5,33) =16.9, Left Stride Length; F (5,33) =15. 

***Pallidal local EEG and its frequency band’s powers***

**Figure 3:**
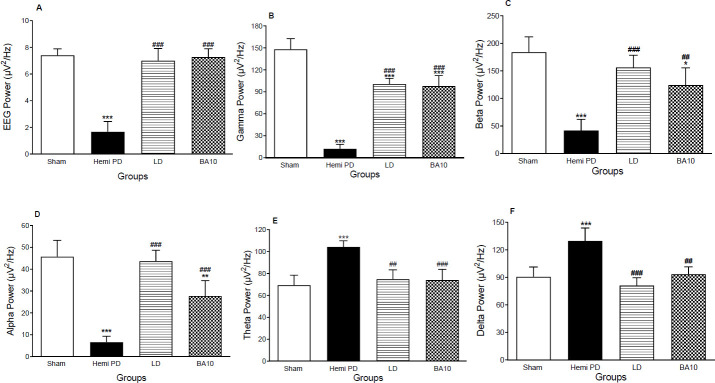
Effects of LD & BA on Pallidal local EEG (A), Gamma power (B), Beta power (C), Alpha power (D), Theta power (E), and Delta power (F). Data were shown as mean ± SEM (n=8) and analyzed using one-way ANOVA and Tukey's *post hoc* tests to compare various groups. *P< 0.05, **P<0.01, and ***P<0.001 compared with the sham animals. #*P*<0.05, ##*P*<0.01, and ###*P*<0.001 vs hemi PD, three weeks after surgery. (Hemi PD=Lesioned animals, LD=L-dopa 20 mg/kg treated group, BA0.5, BA5, BA10 = Betulinic acid 0.5, 5, and 10 mg/kg treated groups)

Animals with MFB-lesion indicated a significant decrease in the GP local EEG power in comparison with the sham group (*P*<0.001). BA (10 mg/kg) and LD (20 mg/kg) significantly increased the EEG power (*P*<0.001, Figure 3A), F (3,12) =74.6, versus hemi PD group. Similarly, gamma and beta powers were dramatically reduced in hemi PD rats versus the sham group, (*P*<0.001). Treatment of hemi PD animals with LD (20 mg/kg) and BA (10 mg/kg) considerably boosted gamma power (*P*<0.001 for each one, Figure 3B), F (3,12) =90, and beta power (*P*<0.001 & *P*<0.01 respectively, Figure 3C), F (3,12) =23.1, versus hemi PD group. Alpha power also showed a significant reduction in hemi PD animals in comparison with the sham group (*P*<0.001), however after LD and BA treatments a remarkable increase in alpha power was observed (*P*<0.001, Figure 3D) F (3,12) =43.3. Conversely, a noticeable increase in theta and delta powers was found after the MFB lesion (*P*<0.001) versus the sham group which was inverted by LD and BA treatment (*P*<0.01 & *P*<0.001 respectively, Figure 3E), F (3,14) =14.6, and (*P*<0.001 & *P*<0.01 respectively, Figure 3F), F (3,13) =16.5.

***Biomarkers of oxidative stress and serum dopamine alterations***

*GSH and Catalase activity*

**Figure 4.**
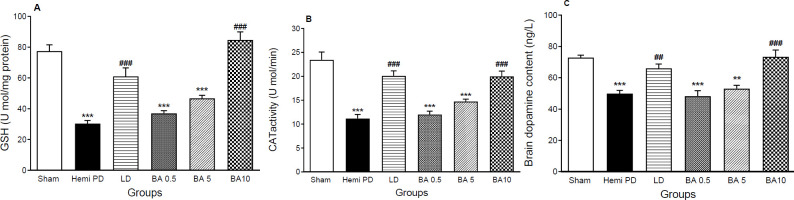
Effects of BA and LD on the concentration of GSH (A), catalase (B), and dopamine levels. Data were shown as mean ± SEM (n=8) and analyzed using one-way ANOVA and Tukey's *post hoc* tests to compare various groups, **P<0.01 & ***P<0.001 compared with the sham group. ##*P*<0.01 & ###*P*<0.001 compared with hemi PD animals, three weeks after surgery. (Hemi PD=lesioned animals, LD=L-dopa 20 mg/kg treatment group, BA0.5, BA5, BA10 = Betulinic acid 0.5, 5, and 10 mg/kg treated groups)

Endogenous regulatory molecules were assessed to evaluate the connection among neurons. Consequently, it revealed that the activities of GSH and CAT were drastically decreased (*P<0.001*) in the hemi PD group in comparison with the sham rats. Treatment with LD (20 mg/kg) and BA (10 mg/kg) significantly reversed the activity of GSH in LD (*P*<0.001, Figure 4A) and BA groups (*P*<0.001, Figure 4A), F (5,26) =52.6. The activity of CAT in both LD and BA treatment groups noticeably reversed compared with the hemi PD group as well (*P*<0.001, Figure 4B) F (5,33) =24.7. No meaningful differences were found in either GSH or CAT activities between BA (0.5 & 5 mg/kg) and hemi PD groups. 

*Brain dopamine concentration*

Evaluating the concentration of DA showed that 6-OHDA induced a reduction in the levels of DA in the brain of hemi PD rats in comparison with the sham groups (*P*<0.001). Treatment with LD (20 mg/kg) and BA (10 mg/kg) for 7 days attenuated 6-OHDA-induced DA reduction in hemi PD groups (*P*<0.01 & *P*<0.001 respectively, Figure 4C), F (5,21) =15.5. No significant differences were shown in DA concentration between lower doses of BA (0.5 & 5 mg/kg) and the hemi PD group. 

## Discussion

The present study revealed the antioxidant potential of BA in the rat model of 6-OHDA-induced neurotoxicity. We demonstrated that chronic administration of BA dramatically improves signs of neurotoxicity in motor dysfunction, pallidal local EEG frequency bands’ power, and refinement in the level of GSH, catalase activity, and DA concentration. In our experiments, BA showed a dose-dependent manner, and its most effective dose was 10 mg/kg. It was recognized that in some behavioral assessments, it was even more effective than L-Dopa which is the standard and placed in the first line of drugs for PD and is introduced as the best choice to treat Parkinsonism. 

Behavioral symptoms are one of the features of PD. They are associated with a reduced quality of life and increased trouble for caregivers **(**19**)**. It has been realized that MFB lesioning led to an elevated contralateral rotation and motor function deficit. This phenomenon is related to terminal degradation, neuronal death, and specific events in the basal forebrain **(**29**)**. Similarly, in our experiment, the rotational behavior was evaluated (two weeks after surgery) using the apomorphine test, to prove the physiological impairments associated with PD, and to confirm the creation of hemi parkinsonian rats in different groups. The final assessments were conducted one week following the treatment by BA and LD (the treatment lasted for one week after confirming the hemi PD-model by apomorphine). Our findings showed the ability of BA, as a potent therapeutic agent against 6-OHDA-induced neurotoxicity that exerts neuroprotective effects. 

Regarding the severe side effects of long-term use of L-Dopa which leads to dyskinesia, abnormal movements, and gastrointestinal disorders **(**30**)**, recent research suggests the phytochemical compounds such as ellagic acid, gallic acid, curcumin, and chrysin with neuroprotective capabilities, as substantial sources in the prevention or even treatment of PD **(**31**)**. 

BA is a lupine-type five-ring three-stock soap existing in the bark of various natural plants, mainly in the Birch (genus Betula) (32). Various pharmacological potentials of BA, including anti-cancer, antiviral against such as hepatitis B virus (HBV), anti-human immunodeficiency virus (HIV), anti-inflammatory and immune regulatory(33), and protective antioxidant potential against cerebral ischemia-reperfusion injury have been reported in different studies (34). The main consequences of oxidative stress are behavioral and memory impairments. Some investigations have shown that pretreatment with BA in AD animals, improved cognitive and neuro-behavioral impairments (35). Thus, we assessed the motor functions of the animals via Stride length and Morpurgo’s tests and proved the efficacy of BA on the rats’ movement.

Moreover, in this study, following the 6-OHDA administration in MFB, a significant decrease in the brain GSH content and brain DA level, as well as CAT activity lowering, were observed which were converted by the administration of BA. Reduction in the concentration of total GSH and antioxidant enzymes, following 6-OHDA administration was associated with motor impairment, which was observed by an elevation in the number of apomorphine-induced rotations. Different studies reported that activation of the apoptosis pathways is related to oxidative stress **(**36**)**. DA neurons are more susceptible to oxidative stress which can be produced by ROS generation and inflammatory mediators generated by 6-OHDA-induced in Hemi PD rats **(**37**)**. These factors are the primary mediators of neuronal apoptosis and cell death (38). 

CAT is a crucial antioxidant enzyme that scavenges free radicals and is closely related to aging and death (39). Shreds of evidence indicated that oxidative stress and increased H_2_O_2_ levels lead to increased CAT, presumably as a compensatory response to counteract H_2_O_2_-mediated oxidative damage (40).

It has been reported that the ventral midbrain level of GSH, decreases in PD patients, and during the PD progression. GSH depletion was mentioned as the primary indicator of oxidative stress, even before the occurrence of other PD hallmarks (41).

 The failure of GSH-associated detoxification relating to an increase in the DA turnover could raise the basal production of hydrogen peroxide and subsequently GSH stocks depletion (42).

Previous studies reported that BA could dramatically decrease the LPO levels while increasing GSH and CAT enzymes’ activities in the thymus, proving the protective role of BA against oxidative-associated damage to the lymphoid immune organ (43). BA has also been shown to inhibit splenocyte apoptosis by up-regulating antioxidant enzymes, decreasing LPO, restoring mitochondrial function, and regulating the mitochondrial signaling pathway (44). Additionally, a survey confirmed that BA in combination with vitamin E could restore the T-2 toxin-triggered intestinal barrier dysfunction via regulating CAT, GSH, and MDA levels and inhibiting the inflammatory responses (45). Consistent with the above reports and our other research which revealed that BA administration could significantly increase the content of GPX, MDA, and SOD **(**30**)**, the current study showed that BA enhanced the concentration of GSH in the rat brains and is a potent antioxidant as evident by preventing CAT inhibition. 

Concerning the field of electrophysiological potential (local EEG) which was recorded from GP, various studies exactly described the main motor dysfunction-associating electrophysiological phenomena in PD, including bradykinesia, tremor, and dyskinesias (46). Different electrophysiological symptoms, e.g., variations of EEG-detected background activity and complicated problems in functional connectivity on the cortico-subcortical and cortico-cortical levels, are linked to PD (47). To investigate the role of the basal ganglia (BG) in specific motor performance, these recordings could potentially help to study the frequency bands’ power of the EEG within different kinds of movements (3). In this study, GP local EEG amplitude, was dramatically reduced in the non-treated group after lesioning MFB, and it reversed following LD and BA treatment, however, there were some variations in different bands’ power that will be described below.

In PD, dopaminergic prescription enlarged gamma activity resulting in motor function improvement, hence it can be proposed that a flat in the progress of motor tasks could result from the synchronized activity of the gamma band in BG (5, 48). In the cortico-basal ganglia loops, beta and gamma range oscillations are considered important factors in motor control (6). The most important result leading to progress in PD therapy is beta hypersynchrony in motor circuits **(**46**)**. The net DA levels at the cortical input to the BG could modulate the oscillatory beta activity and facilitate the suitable production of action potentials. Thus, decreased level of DA impairs this key function (49). Our results displayed that gamma and beta powers were reduced in MFB-lesioned animals and the treatment with LD and BA could significantly elevate their frequency bands’ power.

LD intake regulates scalp EEG activity in patients with PD and is closely connected to the power elevation in the alpha and beta rhythms in centroparietal sites (50). It has been found that there is a drastic reduction in the peak of the alpha wave in PD patients in comparison with healthy subjects. In addition, it has been revealed that the theta and delta bands increase with aging (51). The delta band is linked to sleep modulation, and sleep disorders could be connected with delta band disruption. Surprisingly, sleep disorders are considered one of the most accepted symptoms of PD (4). Moreover, an increase in delta activity and a decrease in alpha power have been found in PD patients with dementia when compared with PD patients without dementia (47). Additionally, a rise in the power of the theta band and a decrease in the power of beta and gamma bands have been also displayed in PD patients in comparison with healthy individuals (52). Similarly, we demonstrated a remarkable increase in the level of alpha, beta, and gamma powers, and a decrease in theta and delta powers after prescribing LD and BA. The antioxidant nature of BA in improving motor function and boosting the level of antioxidant enzymes in 6-OHDA-induced rats is similar to other antioxidant substances including gallic acid, curcumin, and chrysin, which has been confirmed in several studies **(**3, 31, 53**)**, however, when it comes to subdivisions of EEG power, there have been inconsistencies among BA and some antioxidant substances. For instance, the treatment with gallic acid has been found to not be effective on beta and alpha bands **(**53**)**. Also, ellagic acid, was not able to reverse the alpha and delta bands in Hemi-PD rats, while BA turned all of them back **(**3**)**.

## Conclusion

Our findings demonstrated the antioxidant role of BA on brain oxidative damage by 6-OHDA, improving animals’ motor behaviors and boosting the level of dopamine and antioxidant enzymes. Moreover, the potential role of BA as a post-lesion therapeutic agent on muscle stiffness and GP local EEG amplitude was confirmed, indicating the probable potential effect of BA in the prevention and treatment of PD. Therefore, this study suggests that the triterpenoid BA administration can attenuate the neurotoxin-induced motor impairment, EEG disturbances, and inhibition of oxidative stress in hemi parkinsonian rats. However, further investigation is required to clarify the specific mechanisms associated with these results. 

## Authors’ Contributions

All authors contributed in (a) conception and design, or data analysis and interpretation; (b) preparing the manuscript or revising, and (c) preparing the final version. MA contributed to the electrophysiological data analysis, making the model of Parkinson’s disease, performing behavioral tests, and preparing the manuscript. AS contributed to protocol design, supervising the study process, and monitoring the electrophysiological records. YF contributed to the monitoring and approval of behavioral tests in animals.

## Ethical Information

We confirm that this work is original and has not been published elsewhere, nor it is currently under consideration for publication elsewhere. In addition, all experiments were approved by the Ethics Committee of Ahvaz Jundishapur University of Medical Sciences (IR.AJUMS.REC.1396.662). 

## Conflicts of Interest

No conflicts of interest to declare.
